# Expressions of Serum lncRNAs in Diabetic Retinopathy – A Potential Diagnostic Tool

**DOI:** 10.3389/fendo.2022.851967

**Published:** 2022-04-07

**Authors:** Saumik Biswas, Ali Coyle, Shali Chen, Miso Gostimir, John Gonder, Subrata Chakrabarti

**Affiliations:** ^1^Department of Pathology and Laboratory Medicine, Western University, London, ON, Canada; ^2^School of Biomedical Engineering, Western University, London, ON, Canada; ^3^Department of Ophthalmology, Western University, London, ON, Canada

**Keywords:** lncRNA, epigenetics, diabetic retinopathy, biomarker, diagnosis

## Abstract

With increasing incidence of diabetes worldwide, there is an ever-expanding number of patients with chronic diabetic complications such as diabetic retinopathy (DR), one of the leading causes of blindness in the working age population. Early screening for the onset and severity of DR is essential for timely intervention. With recent advancements in genomic technologies, epigenetic alterations in DR are beginning to unravel. Long non-coding RNAs (lncRNAs), which are key epigenetic mediators, have demonstrated implications in several (DR) related processes. Based on the previous research, we have developed a serum-based, multi-panel PCR test using 9 lncRNAs (*ANRIL*, *MALAT1*, *WISPER*, *ZFAS1*, *H19*, *HOTAIR*, *HULC*, *MEG3*, and *MIAT*) to identify and validate whether this panel could be used as a diagnostic and prognostic tool for DR. We initially used a cell culture model (human retinal endothelial cells) and confirmed that 25 mM glucose induces upregulations of *ANRIL*, *HOTAIR*, *HULC*, *MALAT1*, and *ZFAS1*, and downregulation of *H19* compared to 5 mM glucose controls. Then as an initial proof-of-concept, we tested vitreous humor and serum samples from a small cohort of non-diabetic (N=10) and diabetic patients with proliferative retinopathy (PDR, N=11) and measured the levels of the 9 lncRNAs. Differential expressions of lncRNAs were found in the vitreous and serum of patients and showed significant correlations. We expanded our approach and assessed the same lncRNAs using samples from a larger cohort of diabetic (n= 59; M/F:44/15) and non-diabetic patients (n= 11; M/F:4/7). Significant increased lncRNA expressions of *ANRIL*, *H19*, *HOTAIR*, *HULC*, *MIAT*, *WISPER* and *ZFAS1* were observed in the serum of diabetic patients (with varying stages of DR) compared to non-diabetics. No significant correlations were demonstrated between lncRNA expressions and creatinine or glycated hemoglobin (HbA1C) levels. Using ROC and further analyses, we identified distinct lncRNA phenotype combinations, which may be used to identify patients with DR. Data from this study indicate that a panel of serum lncRNAs may be used for a potential screening test for DR. Further large-scale studies are needed to validate this notion.

## Introduction

Diabetes mellitus (DM) continues to wreak havoc across the world, where 463 million of the global population are estimated to have DM, while 700 million are projected to have DM by 2045 (1). DM is characterized by chronic hyperglycemia and the sustained elevation of blood glucose levels promotes cellular and tissue damage, which consequently leads to the development of diabetic complications. Among these complications, diabetic retinopathy (DR) is a prevalent microvascular complication of DM and one of the leading causes of preventable blindness in the working age population (2). To prevent irreversible visual loss from DR, screening for the onset and severity of DR has become critical for timely and necessary ophthalmic examination and treatment (3). To bridge this gap in healthcare delivery, here we describe a novel lncRNA-based serum test which may facilitate effective screening and identification of DR.

According to the International Council of Ophthalmology classification, DR can be categorized into five progressive stages of retinopathy: no retinopathy, mild non-proliferative, moderate non-proliferative, severe non-proliferative, and proliferative retinopathy (4). Each stage of DR is categorized by distinct clinical observations, which can include retinal vascular-related abnormalities (microaneurysms, intraretinal hemorrhages, and venous beading), retinal thickening (edema), accumulation of lipid deposits, presence of neovascularization, and/or vitreous/preretinal hemorrhage (4). According to one study in the US involving 4617 patients, 6.6% of patients with non-proliferative DR (NPDR) progressed to proliferative DR (PDR). Proliferative DR is considered the more advanced stage of DR and is characterized by the onset of neovascularization at various retinal regions (including new vessels at the inner surface of the retina, on or near the optic disc and new vessels elsewhere in the retina) compared to non-proliferative stages of DR (4, 5). These vessels are prone to bleeding and may undergo fibrosis and contraction, which can ultimately lead to blindness and a profound impact on the quality of life for patients (6).

Presently, there are several relevant risk factors that are used to monitor potential development of DR, including the duration of diabetes, poor glycemic control (high glycated hemoglobin [HbA1c levels]), presence of hypertension, dyslipidemia, and a higher body mass index (7). However, based on several large population-based clinical studies, substantial variation in the onset and severity of DR have been documented in patients living with DM that is not completely explained by the known risk factors. For example, total glycemic exposure (duration of diabetes and HbA1c values) explained only ~11% of the variation in DR risk for the entire study population in the Diabetes Control and Complications Trial, which suggests that the remaining 89% of the variation risk is due to other factors (i.e., genetic determinants and/or epigenetic factors) in the diabetic milieu that are independent of HbA1c (8). As a result, to effectively monitor glycemic control and implement timely treatment protocols, it becomes imperative that research efforts are invested towards identifying these unexplained factors and novel molecular players that continue to contribute to the development and progression of microvascular complications such as DR. Furthermore, implementation of a test to effectively identify and treat patients early in the disease process will result in considerable cost savings, since the cost of caring for patients with PDR is much greater than the costs of managing patients with NPDR (9, 10).

Given the advent of novel genomic technologies within the last decade, recent studies examining the epigenetic landscape in DR have shed light on the ‘dark matter’ of the epi/genome and have opened the door for the selective targeting of promising molecular candidates in the diagnosis and/or therapy of DR. Among these molecules, long non-coding RNAs (lncRNAs), which do not have protein-coding potential and are greater than 200 nucleotides in length, have demonstrated critical implications in a number of DR-related processes including angiogenesis (11–13), inflammation (14), and even fibrosis-related mechanisms (i.e., endothelial-to-mesenchymal transition) (15).

In this study, based on the collective findings from our previous lncRNA microarray involving human retinal endothelial cells (HRECs) subjected to basal and high glucose conditions (GEO ID: 122189) and existing mechanistic-based research performed by us and others (11–19), we selected 9 prominent lncRNAs (*ANRIL*, *MALAT1*, *WISPER*, *ZFAS1*, *H19*, *HOTAIR*, *HULC*, *MEG3*, and *MIAT*) and developed a multi-lncRNA PCR panel in order to investigate and validate whether a diagnostic biomarker panel could be used to distinguish between patients with non-DR and DR. We then carried out additional statistical analyses to determine the potential discriminative ability of this lncRNA-panel to diagnose and identify the various sub-stages of DR.

## Materials and Methods

### Development of the Multi-lncRNA PCR Panel

Customized human lncRNA primers were developed (**Table 1**) and subsequently aliquoted and air dried into a 96-well plastic qPCR plate. Custom qPCR plates were stored in -20°C prior to use. A single panel (consisting of 10 wells) in the lncRNA PCR plate examined 9 distinct lncRNAs (*MALAT1, HOTAIR, H19, MEG3, ANRIL, MIAT, WISPER, ZFAS1*, and *HULC*) and one house-keeping gene (β-actin). Synthesized cDNA (following the protocol below) was diluted and combined with SYBR-green master mix (Takara Bio, Mountain View, CA, USA), and then aliquoted into the 96-well PCR plate containing the pre-aliquoted PCR panel. The panel was then inserted into the LightCycler 96 System (Roche Diagnostics, Laval, QC, CAN) for amplification.

**Table 1:** Human lncRNA qPCR primers used for the PCR panel.

Target Gene (Human):	Oligonucleotide Sequence (5’→3’):
*ACTB (B-actin)*	F: CCTCTATGCCAACACAGTGC
	R: CATCGTACTCCTGCTTGCTG
*HOTAIR*	F: GGGGCTTCCTTGCTCTTCTTATC
	R: CTGACACTGAACGGACTCTGTTTG
*H19*	F: AAAGACACCATCGGAACAGC
	R: AGAGTCGTCGAGGCTTTGAA
*WISPER*	F: CCATCTGTGGGACATCTGTG
	R: TGGGGGCTGTGGAGATAGTA
*ZNFX1-AS1 (ZFAS1)*	F: CAGCGGGTACAGAATGGA
	R: TCAGGAGATCGAAGGTTGTAGA
*HULC*	F: ATCTGCAAGCCAGGAAGAGTC
	R: CTTGCTTGATGCTTTGGTCTGT
*ANRIL*	F: CCCTTATTTATTCCTGGCTCC
	R: GACCTCGCTTTCCTTTCTTCC
*MALAT1*	F: TCTTAGAGGGTGGGCTTTTGTT
	R: CTGCATCTAGGCCATCATACTG
*MIAT*	F: GGGAGGGGAAATGGGTGATGTA
	R: TAACGCCAAATGTGAAGTGTGA
*MEG3*	F: CGGCTGGGTCGGCTGAAGAACT
	R: CCGCCCAAACCAGGAAGGAGAC

### RNA Isolation and Quantitative Real-Time Polymerase Chain Reaction (RT-qPCR)

As described by us previously (12–15), total RNA was extracted using the TRIzol reagent (Invitrogen). RNA concentrations were quantified using a spectrophotometer (260 nm; Gene Quant, Pharmacia Biotech, USA) and 1-2 μg of total RNA was reverse transcribed to complementary DNA (cDNA) using a high-capacity cDNA reverse-transcription kit (Applied Biosystems/Thermo Fisher Scientific). cDNA was then amplified in the LightCycler 96 System (Roche Diagnostics, Laval, QC, CAN) using the multi-lncRNA PCR panel. RT-qPCR results were analyzed using the LightCycler 96 SW 1.1 software (Roche) and expression levels were calculated by the relative standard curve method using β-actin as an internal control for sample normalization.

### Cell Culture

Prior to the clinical validation of our panel, we first validated our assay *in vitro*. Human retinal microvascular endothelial cells (HRECs; Cell Systems, Kirkland, WA, USA; catalog number ACBRI 181) were cultured in endothelial basal media-2 (EBM-2, Lonza, Walkersville, MD, USA) containing endothelial growth media-2 (EGM-2) SingleQuots (Lonza). All cells were grown in 75 cm^2^ culture flasks and maintained in a humidified incubator containing 5% CO_2_ at 37°C. As described previously (12, 14), to reduce variability for experimentation, cells were used between passages three and six and the cellular densities were determined accordingly based on the type of culture plates used for each experiment. Generally, once 80% confluence was obtained post-seeding, ECs were cultured in serum and growth factor-free medium overnight before exposure to different D-glucose levels (final glucose concentrations of 5 mmol/L, mimicking normoglycemia [NG], and 25 mmol/L, mimicking hyperglycemia [HG]) for 48 hours; the selected glucose level is based on a large volume of previous experiments (12–15). Twenty-five mmol/L L-glucose was used as osmotic control. All *in vitro* experiments were independently repeated at least three times and performed with six replicates, unless specified.

### Collection and Analyses of Vitreous and Serum Samples

The study was approved by the Western Research Ethics Board and Lawson Health Research Institute at the University of Western Ontario (London, ON, CAN). Informed consent was received from patients prior to obtaining specimens and the research was conducted following the *Declaration of Helsinki*. As an initial test to identify whether serum lncRNAs appropriately reflect the vitreous lncRNAs, we collected both serum and undiluted vitreous humor (VH) samples from patients, undergoing a pars plana vitrectomy by an experienced vitreoretinal surgeon (12). These samples were categorized into two groups: control and DR.

In the initial cohort, the DR group comprised of patients diagnosed with advanced stages of DR, including proliferative DR (PDR; n= 11; mean age ± SD= 60.9 ± 10.4 years; 10 males and 1 female, 1 type I diabetic and 10 type 2 diabetics), while the control group consisted of patients that had no previous history of DR and were diagnosed with idiopathic macular hole or a separate non-diabetic ocular condition (n= 10; mean age ± SD= 69.2 ± 8.9 years; 2 males and 8 females). PDR was defined as the presence of neovascularization or fibrous proliferation of the disc or elsewhere on the retina. Total RNA was extracted from 500 μL of VH and 200 μL of serum samples using a serum RNA extraction kit (Bio Basic Inc., Markham, ON, CAN) and was subsequently examined by RT-qPCR.

Following the results of our initial experiment, we expanded our test using only serum samples from a larger new cohort of patients. Briefly, undiluted serum samples were collected in BD gold-top serum separator tubes. Serum specimens were submitted to the research laboratory, where total RNA was extracted from 200 μL of serum samples using TRIzol and the serum RNA extraction kit mentioned above. The expression levels of the 9-specific lncRNAs (*ANRIL*, *H19*, *HOTAIR*, *HULC*, *MALAT1*, *MEG3*, *MIAT*, *WISPER* and *ZFAS1*) were assessed using RT-qPCR, where a research technician was masked to the sample type. The subjects include 7 type I diabetic and 52 type 2 diabetics. Specimens were categorized into two groups: control (‘C’) and diabetic retinopathy (‘P’; DR). The ‘P’ group comprised of diabetic patients diagnosed with varying stages of DR (indicated as ‘0’= diabetic patients with no retinopathy, ‘1’ = diabetic patients with non-proliferative DR [NPDR], and ‘2’= diabetic patients with proliferative DR [PDR]) (n= 59; mean age ± SD= 64.2 ± 11.5 years; 44 males and 15 females), while the control group consisted of non-diabetic patients who were seen at the eye clinic for conditions unrelated to diabetes (n= 11; mean age ± SD= 62.9 ± 17.3 years; 4 males and 7 females).

### Statistical Analyses

Statistical differences were evaluated between groups using GraphPad Prism 7 (La Jolla, CA, USA) and Microsoft Excel (Washington, USA). Data were considered statistically significant if the *p* value was less than 0.05. Statistical significance for the clinical samples (with non-normal distribution) were identified using the Mann-Whitney U test (when comparing two conditions) or Kruskal-Wallis one-way ANOVA (for multiple group comparisons) and linear regression analyses wherever appropriate. ROC curves were used to elucidate clinical significance of lncRNA molecules in control versus diabetic patients with no retinopathy, NPDR and PDR by comparing sensitivities and false positive rates. AUC numbers were approximated simply by sum of the areas of rectangles in the ROC curves. Creatinine and HbA1c levels were compared between control and patient groups using Spearman’s rank correlation coefficient. Two outliers from control samples I6 and A1 for *MEG3* (86096.52 and 2022.94, respectively) were removed for better graphical representation of the results.

### Data Availability

All data generated or analyzed during this study are included in this published article.

## Results

### Glucose Causes Differential Expressions of lncRNAs in Retinal Endothelial Cells

**Figure 1:**
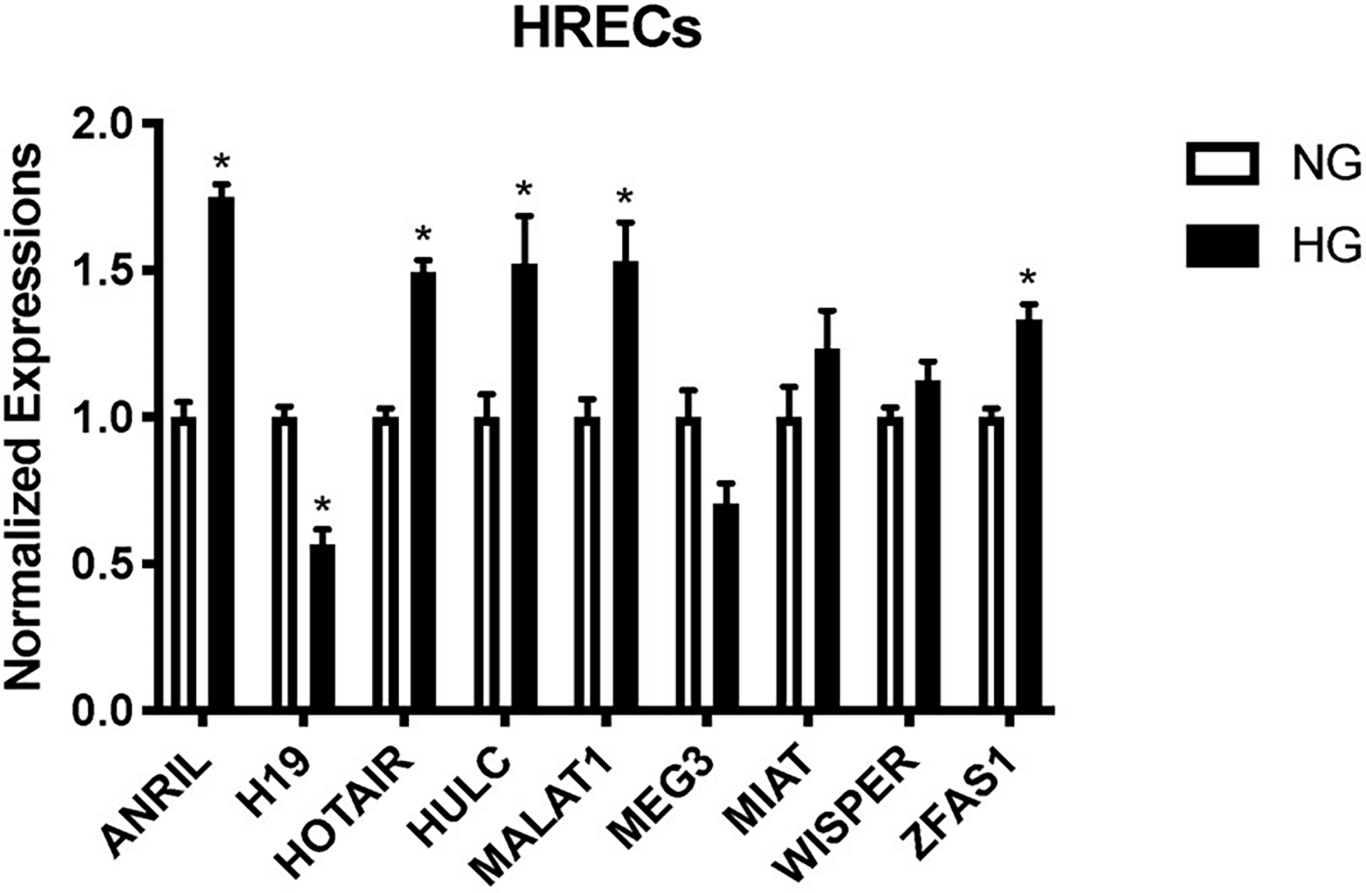
Glucose-treated human retinal endothelial cells (HRECs) demonstrate differential expressions of lncRNAs at 48 hours. RT-qPCR analysis of *ANRIL*, *H19*, *HOTAIR*, *HULC*, *MALAT1*, *MEG3*, *MIAT*, *WISPER* and *ZFAS1* expressions in HRECs exposed to 25 mM (HG) or 5 mM (NG) glucose over 48 hours [data (mean ± SEM); N=6 per group; normalized to β-actin and expressed as a fold change of NG; *p<0.05 compared to NG].

Using our established cell culture model and RT-qPCR (12–15, 17), we confirmed the lncRNA expressions of *ANRIL*, *H19*, *HOTAIR*, *HULC*, *MALAT1*, *MEG3*, *MIAT*, *WISPER*, and *ZFAS1* in HRECs exposed to 25 mM (HG) or 5 mM (NG) glucose over 48 hours from our array (**Figure 1**). As depicted in **Figure 1**, HG induced significant upregulations of *ANRIL*, *HOTAIR*, *HULC*, *MALAT1*, and *ZFAS1*, and a significant downregulation of *H19* in HRECs compared to NG controls. Although not statistically significant, in comparison to NG controls, general trends of upregulation were observed for *MIAT* and *WISPER*, while *MEG3* appeared to demonstrate decreased expressions following HG stimulation. No alterations of such lncRNA expressions were seen when the cells were exposed to 25mM L-glucose (not shown).

### Expressions of lncRNAs in the Serum Correlates With Vitreous Levels in Diabetic Retinopathy

**Figure 2:**
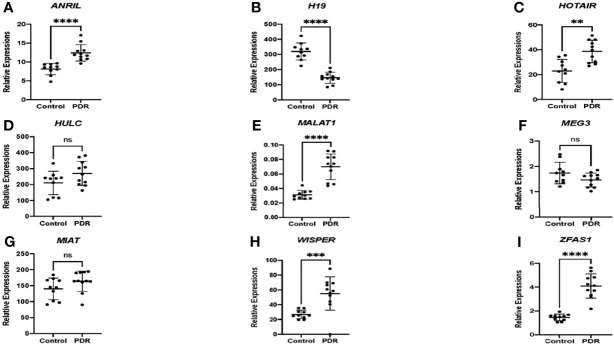
Differential expressions of lncRNAs in the serum of patients. RT-qPCR analyses of **(A)** *ANRIL*, **(B)** *H19*, **(C)** *HOTAIR*, **(D)** *HULC*, **(E)** *MALAT1*, **(F)** *MEG3*, **(G)** *MIAT*, **(H)** *WISPER*, and **(I)** *ZFAS1* expressions in the serum of patients with proliferative diabetic retinopathy (PDR) compared to controls (non-diabetic patients). Data is expressed as a ratio to β-actin. Statistical significance was assessed using the Mann-Whitney U test. Data represents the mean ± SD (N=10 per control group or N=11 per PDR group; ns, not significant, **p<0.01, ***p<0.001, or ****p<0.0001).

**Figure 3:**
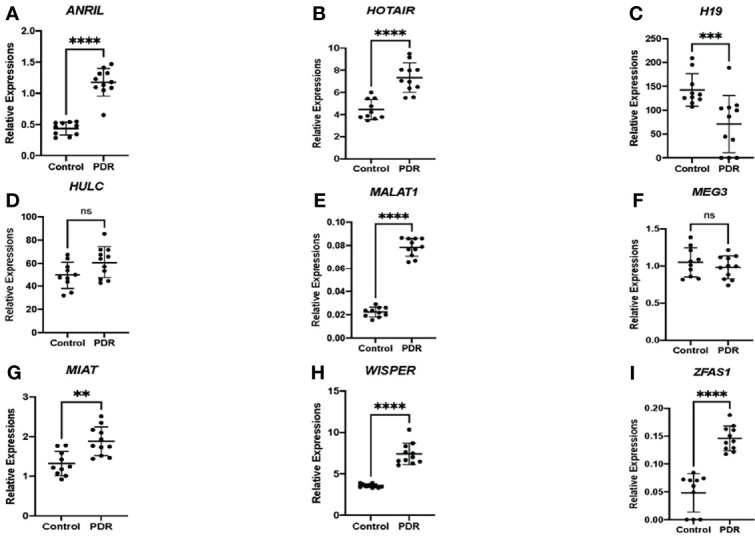
Differential expressions of lncRNAs in the vitreous fluid of patients. RT-qPCR analyses of **(A)** *ANRIL*, **(B)** *HOTAIR*, **(C)** *H19*, **(D)** *HULC*, **(E)** *MALAT1*, **(F)** *MEG3*, **(G)** *MIAT*, **(H)** *WISPER*, and **(I)** *ZFAS1* expressions in the vitreous of patients with proliferative diabetic retinopathy (PDR) compared to controls (non-diabetic patients). Data is expressed as a ratio to β-actin. Statistical significance was assessed using the Mann-Whitney U test. Data represents the mean ± SD (N=10 per control group or N=11 per PDR group; ns, not significant, **p<0.01, ***p<0.001, or ****p<0.0001).

As an initial proof-of-concept experiment, we wanted to determine whether the selected lncRNAs could be detected in human DR. So, we analyzed both vitreous humor and serum samples from an initial small cohort of patients as outlined above using the customized qPCR-based panel. Similar to the trends observed *in vitro*, differential expressions of lncRNAs were found in the vitreous and serum of patients with PDR compared to non-PDR patients—further suggesting the biological significance of lncRNAs in human DR (**Figure 2**). Specifically, alterations were significantly pronounced in the serum expression levels of *ANRIL* (**Figure 2A**), *H19* (**Figure 2B**), *HOTAIR* (**Figure 2C**), *MALAT1* (**Figure 2E**), *WISPER* (**Figure 2H**) and *ZFAS1* (**Figure 2I**) in PDR. However, we did not find significant alterations with respect to the serum expressions of *HULC* (**Figure 2D**), *MEG3* (**Figure 2F**) and *MIAT* (**Figure 2G**) in PDR, and such findings may be related to the relatively small sample size. Moreover, as shown in **Figure 3**, significant expressions of *ANRIL* (**Figure 3A**), *HOTAIR* (**Figure 3B**), *H19* (**Figure 3C**), *MALAT1* (**Figure 3E**), *MIAT* (**Figure 3G**), *WISPER* (**Figure 3H**) and *ZFAS1* (**Figure 3I**) were observed in the vitreous of PDR patients, while statistical significances were not observed for *HULC* (**Figure 3D**) and *MEG3* expressions (**Figure 3F**).

**Figure 4:**
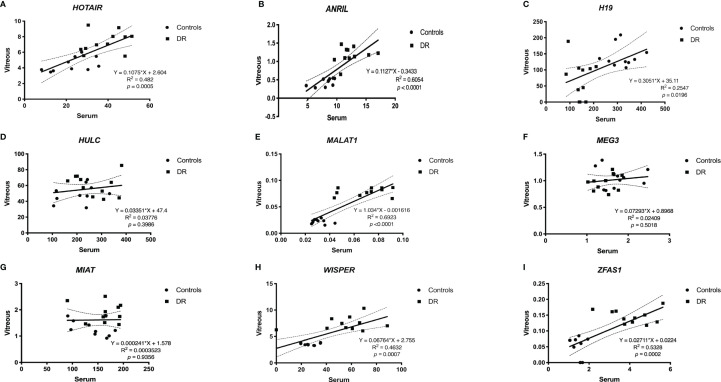
Pearson correlation analyses between serum and vitreous samples. When comparing between serum and vitreous samples, significant correlations were observed for **(A)** *ANRIL*, **(B)** *H19*, **(C)** *HOTAIR*, **(E)** *MALAT1*, **(H)** *WISPER* and **(I)** *ZFAS1*, which suggests that the expressions of these lncRNAs can be reflected from the serum and vitreous of patients with DR. Although we did find significant correlations between serum and vitreous concentrations of **(D)** *HULC*, **(F)** *MEG3*, and **(G)** *MIAT*, including a larger sample size may further help confirm the relationships between these markers and sample types [*p*<0.05 was considered significant; dotted lines represent 95% confidence intervals. N= 10 for control group and N= 11 for diabetic group; and normalized to β-actin]. DR, diabetic retinopathy.

When comparing the level of lncRNA expressions between serum and vitreous samples, significant correlations were observed for *HOTAIR* (**Figure 4A**), *ANRIL* (**Figure 4B**), *H19* (**Figure 4C**), *MALAT1* (**Figure 4E**), *WISPER* (**Figure 4H**) and *ZFAS1* (**Figure 4I**), which suggests that the level of expression for these lncRNAs in the serum parallels that of the lncRNA expression levels in the vitreous. We did not find significant correlations between serum and vitreous concentrations of *HULC* (**Figure 4D**), *MEG3* (**Figure 4F**), and *MIAT* (**Figure 4G**).

### Demographic Data and Disease Characteristics of the Study Population

Following the results from our proof-of-concept experiment, we expanded our study by examining the expression of the above lncRNAs in the serum of a new cohort of non-diabetic patients and diabetic patients with various stages of DR. As shown in **Table 2**, males were more predominant in the diabetic patient group, while females were more prevalent in the control group. As expected, the diabetic patient group also had a slightly higher means of BMI, hemoglobin A1c [HbA1c], and creatinine levels compared to the control group. However, when examining the diabetic patient groups according to the presence of retinopathy, no significant differences were observed for age, BMI, or creatinine levels, while significances were observed for HbA1c and gender distribution between diabetic patients with retinopathy and those without. Furthermore, when comparing between NPDR and PDR patients, no significant differences were observed across age, type of diabetes, BMI, creatinine, HbA1c, and gender distribution.

**Table 2:** Demographic data and disease characteristics of the study groups.

			Control vs. All Patients				DM vs. DR		Control vs. DR				NPDR vs. PDR	
Variables	Control	All Patients	*p*	OR (95% CI)	DM	DR	*p*	OR (95% CI)	*p*	OR (95% CI)	NPDR	PDR	*p*	OR (95% CI)
Number	11	59			10	49					26	23		
Age														
Mean ± SD	62.9 ± 17.29	64.2 ± 11.49	0.52		70.7 ± 7.13	62.9 ± 11.75	0.9		0.62		65.6 ± 11.16	59.7 ± 11.63	0.9	
≤ 50	3 (27.3)	5 (8.5)	0.07	Reference	0 (0)	5 (10.2)	0.29	Reference	0.13	Reference	1 (3.8)	4 (17.4)	0.12	Reference
> 50	8 (72.7)	54 (91.5)		4.05 (0.81-20.31)	10 (100)	44 (89.8)		0		3.3 (0.65-16.63)	25 (96.2)	19 (82.6)		0.19 (0.02-1.84)
Sex														
Female	7 (63.6)	15 (25.4)	*0.01*	Reference	4 (40)	11 (22.4)	*0.01*	Reference	*0.007*	Reference	5 (19.2)	6 (26.1)	0.6	Reference
Male	4 (36.4)	44 (74.6)		*5.13 (1.32-20.02)*	6 (60)	38 (77.6)		*2.3 (0.55-9.64)*		*6.05 (1.49-24.51)*	21 (80.8)	17 (73.9)		*0.67 (0.18-2.6)*
Other														
BMI (kg/m^2^)	29.3 ± 3.08	31.1 ± 6.29	0.24	NA	30.4 ± 4.53	31.2 ± 6.54	0.4	NA	0.25	NA	31.9 ± 5.56	30.5 ± 7.26	0.9	NA
HbA1c (µmol/L)	5.6 ± 0.74	7.5 ± 2.63	*< 0.001*	*NA*	6.6 ± 1.12	7.7 ± 2.82	*0.02*	*NA*	< *0.001*	*NA*	6.9 ± 2.88	8.3 ± 2.6	0.06	*NA*
Creatinine	75.8 ± 21.58	95.5 ± 48.58	0.1	NA	82 ± 14.71	97.9 ± 52.07	0.25	NA	0.1	NA	101.3 ± 48.68	94.8 ± 54.79	0.8	NA

Data are represented as number (percentage) or mean ± standard deviation (SD). DM, diabetes mellitus patients without retinopathy; NPDR, non-proliferative diabetic retinopathy; PDR, proliferative diabetic retinopathy. OR (95% CI), odds ratio and 95% confidence interval. Mann Whitney u test for quantitative variables and chi-square test for qualitative data were applied. Items in italics indicate p < 0.05. NA, not applicable.

### A Pattern of lncRNA Alterations Were Observed in the Serum in Diabetes

**Figure 5:**
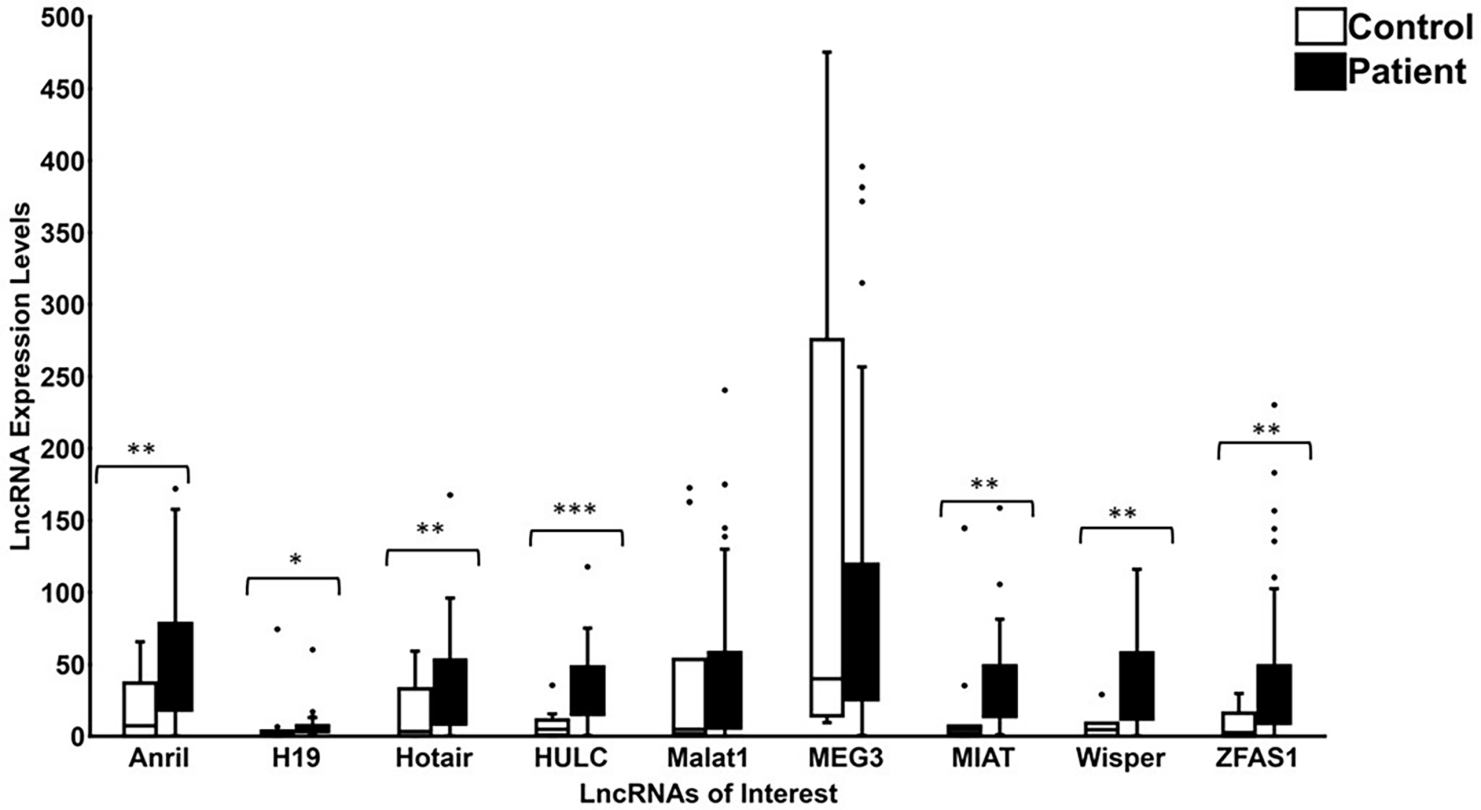
Expression levels of lncRNAs in patients and controls. Boxplots represent comparison between expression level of 9 specific lncRNAs (*ANRIL*, *H19*, *HOTAIR*, *HULC*, *MALAT1*, *MEG3*, *MIAT*, *WISPER* and *ZFAS1*) detected by RT-qPCR in patient and control groups. The median and interquartile range (25^th^ -75^th^ percentile), are shown as solid horizontal lines. Two outliers from control samples I6 and A1 for *MEG3* (86096.52 and 2022.94, respectively) were removed for better graphical representation of the results. Statistical significance was assessed using Mann-Whitney U test (*p<0.05, **p<0.005, or ***p<0.0005; N=11 for control patients and N= 59 for diabetic patients). Statistical significance was considered at *p*<0.05. Legend: Control = Non-diabetic patients without any diabetic retinopathy; Patient = Diabetic patients with or without diabetic retinopathy.

As shown in **Figure 5**, significant increased lncRNA expressions of *ANRIL*, *H19*, *HOTAIR*, *HULC*, *MIAT*, *WISPER* and *ZFAS1* were observed in the serum of diabetic patients (with varying stages of DR; group ‘P’) compared to non-diabetic patients (without DR; group ‘C’). Interestingly, although not significant, *MEG3* lncRNA levels demonstrated decreasing trends in diabetic patients compared to control patients (*p* = 0.67), while *MALAT1* levels exhibited increasing trends in the diabetic patient group (*p* = 0.09). No significant correlations were demonstrated between the lncRNA expression profiles and the creatinine (**Table 3**) or HbA1C levels (**Table 4**) in the non-diabetic and diabetic patient groups.

**Table 3:** Creatinine Spearman’s rank correlation coefficient.

Spearman correlation coefficient ρ (Control n=6, Patient n= 55)									
	ANRIL	H19	HOTAIR	HULC	MALAT1	MEG3	MIAT	WISPER	ZFAS1
Control n = 6									
ρ	-0.45	0.23	-0.42	-0.18	-0.58	0.57	-0.17	-0.40	-0.33
* P*-value	0.224	0.546	0.265	0.637	0.099	0.112	0.668	0.286	0.381
Patient n = 55									
ρ	0.029	-0.080	0.057	0.001	-0.070	-0.112	-0.077	0.040	-0.008
* P*-value	0.836	0.574	0.681	0.996	0.611	0.415	0.576	0.774	0.956
Combined P and C, n=61									
ρ	0.001	0.023	0.000	0.060	-0.135	-0.022	0.005	0.076	-0.024
* P*-value	0.993	0.860	0.998	0.645	0.299	0.864	0.967	0.562	0.851

Spearman's rank correlation coefficients of lncRNAs and Creatinine expression. To obtain p-values, t-statistic was used.

Method:

1. Similar to HbA1c.

**Table 4:** HbA1c Spearman’s rank correlation coefficient.

Spearman's rank correlation coefficient ρ (Control n = 7, Patient n = 51)									
	ANRIL	H19	HOTAIR	HULC	MALAT1	MEG3	MIAT	WISPER	ZFAS1
Control n = 7									
ρ	0.36	-0.49	0.68	0.07	0.00	-0.74	0.14	0.34	0.52
* P*-value	0.427	0.268	0.090	0.878	1.000	0.058	0.758	0.452	0.229
Patient n = 51									
ρ	0.022	0.083	-0.063	-0.044	-0.143	-0.075	-0.054	-0.178	-0.106
* P*-value	0.879	0.564	0.662	0.760	0.318	0.602	0.705	0.211	0.459
Combined P and C, n=58									
ρ	0.237	0.103	0.164	0.167	0.034	-0.157	0.094	0.022	0.133
* P*-value	0.074	0.439	0.219	0.210	0.803	0.238	0.481	0.869	0.320

Spearman's rank correlation coefficients of lncRNAs and HbA1c expression. To obtain p-values, t-statistic was used.

Method:

1. lncRNA and HbA1c amounts for each sample was ranked using RANK.AVG () function.

2. Step 1 was done for control (n=7), for all patients with available HbA1c values (n=51 – some patients missing this information), and for the combination of both (n=58).

3. Correlation between the two ranks for each sample was calculated using CORREL() function.

4. T-statistic, degree of freedom was calculated for each correlation to obtain p-value using TDIST() function.

### Stage Dependent Alterations of lncRNAs in DR

**Figure 6:**
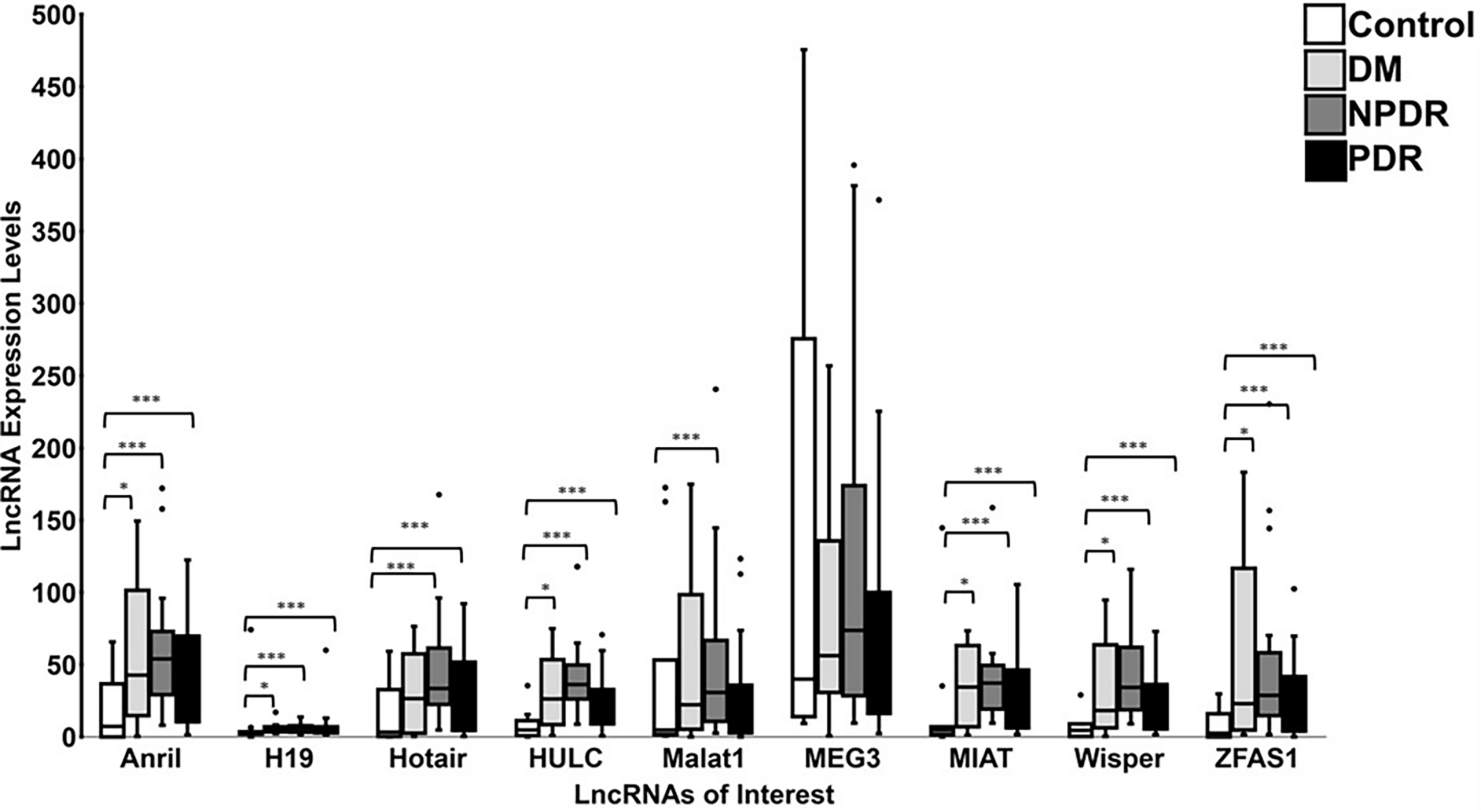
Expression levels of lncRNAs in controls and patients diagnosed with varying stages of DR. Boxplots represent comparison between expression level of 9 specific lncRNAs (*ANRIL*, *H19*, *HOTAIR*, *HULC*, *MALAT1*, *MEG3*, *MIAT*, *WISPER* and *ZFAS1*) detected by RT-qPCR in control and patient groups (DM = diabetic patients with no retinopathy, NPDR = diabetic patients with non-proliferative DR, and PDR = diabetic patients with proliferative DR). The median and interquartile range (25^th^ -75^th^ percentile), are shown as solid horizontal lines. Two outliers from control samples I6 and A1 for *MEG3* (86096.52 and 2022.94, respectively) were removed for better graphical representation of the results. Statistical significance was assessed using Mann-Whitney U test (*p<0.05, ***p<0.0005; N=11 for control patients, N=10 for DM patients, N=26 for NPDR patients, and N=23 for PDR patients). Statistical significance was considered at *p*<0.05.

When the data of various patient subgroups were analyzed, the average expression levels for 8 lncRNAs were generally increased across all diabetic patient sub-groups (‘DM’, ‘NPDR’, and ‘PDR’) when compared to controls (**Figure 6**). Such alterations were not observed with *MEG3*. Interestingly, when comparing between control and ‘DM’ groups, significances were observed for the lncRNAs *ANRIL, H19, HULC, MIAT, WISPER*, and *ZFAS1*. In addition, when the control group was compared with the ‘NPDR’ sub-group, significances were observed for 8 out of the 9 lncRNAs, apart from *MEG3*. Additionally, comparing the lncRNA expression levels between control and PDR groups also demonstrated statistical significance for *ANRIL*, *H19*, *HOTAIR*, *HULC*, *MIAT*, *ZFAS1*, and *WISPER*, while *MEG3* and *MALAT1* did not show significance. These observations suggest a duration-dependent change in the lncRNA expression profiles in the serum, where the duration of diabetes may reflect distinct lncRNA expression patterns. Collectively, the results suggest that these 9 lncRNAs can be used as biomarkers of DR.

To further examine significance of the serum levels of the analytes, area under the curve (AUC) was calculated. AUC analyses demonstrated that the lncRNAs of interest have potential diagnostic value in discriminating diabetic patient sub-groups (diabetes, NPDR, and PDR) from control non-diabetic patients. (95% CI, two-tailed Z-critical values were used to calculate p-values) (**Table 5**). Specifically, using AUC, comparison between control patients and diabetic patients with no DR demonstrated a significant difference in 9 lncRNAs, where *HULC* had the highest AUC (*p* < 0.05) and *MEG3* had the lowest AUC (*p < 0.05*). Furthermore, when analyzing the AUC values for control and NPDR patients, all 9 lncRNAs demonstrated statistical significance and 7 out of 9 lncRNAs had AUC values greater than 0.7, except for *MEG3* and *MALAT1*. Additionally, comparison of AUC values between control patients and PDR patients demonstrated significance for all the lncRNA markers and 6 out of the 9 lncRNAs had AUC values greater than or equal to 0.7, apart from *H19*, *MEG3* and *MALAT1*. Interestingly, when comparing between diabetic patients with no retinopathy and diabetic patients with DR (NPDR or PDR), all 9 lncRNAs demonstrated AUC values ranging between 0.460 to 0.570 with significant statistical differences. Furthermore, although significant, AUC values for the 9 lncRNAs ranged between 0.290 to 0.420 when comparing between NPDR and PDR patients. Collectively, these results demonstrate that the 9 examined lncRNAs may be used as a prognostic tool to discriminate between non-diabetic and diabetic patients with DR, as well as potentially discriminate various stages of DR (mild NPDR to severe PDR) from diabetic patients without DR.

**Table 5:** Diagnostic performance of lncRNA gene expression.

lncRNA	C vs DM		C vs NPDR		C vs PDR		C vs DR		DM vs NPDR		DM vs PDR		DM vs DR		NPDR vs PDR	
	AUC ± SE	*p*	AUC ± SE	*p*	AUC ± SE	*p*	AUC ± SE	*p*	AUC ± SE	*p*	AUC ± SE	*p*	AUC ± SE	*p*	AUC ± SE	*p*
ANRIL	0.77 ± 0.1	5.2E-13	0.86 ± 0.08	2.7E-27	0.75 ± 0.1	1.6E-14	0.81 ± 0.08	4.2E-21	0.55 ± 0.11	9.5E-07	0.4 ± 0.11	1.7E-04	0.48 ± 0.1	2.4E-06	0.35 ± 0.08	1.5E-05
H19	0.78 ± 0.1	1.1E-13	0.76 ± 0.09	2.5E-15	0.69 ± 0.1	5.7E-11	0.73 ± 0.09	2.7E-14	0.56 ± 0.11	5.7E-07	0.48 ± 0.11	1.8E-05	0.52 ± 0.1	4.9E-07	0.42 ± 0.08	5.4E-07
HOTAIR	0.66 ± 0.12	5.8E-08	0.82 ± 0.08	1.3E-20	0.7 ± 0.1	1.7E-11	0.76 ± 0.09	1.4E-16	0.62 ± 0.11	1.9E-08	0.51 ± 0.11	6.3E-06	0.57 ± 0.1	5.4E-08	0.29 ± 0.07	1.3E-04
HULC	0.81 ± 0.1	4.8E-16	0.94 ± 0.05	3.6E-66	0.81 ± 0.09	9.0E-20	0.88 ± 0.07	3.8E-34	0.61 ± 0.11	3.7E-08	0.44 ± 0.11	5.8E-05	0.53 ± 0.1	3.2E-07	0.3 ± 0.08	8.4E-05
MALAT1	0.65 ± 0.12	2.0E-07	0.69 ± 0.1	2.0E-11	0.54 ± 0.11	9.3E-07	0.62 ± 0.1	7.3E-10	0.54 ± 0.11	1.3E-06	0.39 ± 0.1	2.3E-04	0.47 ± 0.1	3.4E-06	0.33 ± 0.08	3.1E-05
MEG3	0.45 ± 0.13	4.4E-04	0.51 ± 0.11	1.8E-06	0.4 ± 0.1	1.1E-04	0.46 ± 0.1	2.0E-06	0.57 ± 0.11	4.0E-07	0.44 ± 0.11	5.1E-05	0.51 ± 0.1	7.9E-07	0.36 ± 0.08	7.1E-06
MIAT	0.75 ± 0.11	7.6E-12	0.87 ± 0.07	1.7E-30	0.75 ± 0.1	2.9E-14	0.82 ± 0.08	9.1E-22	0.52 ± 0.11	3.3E-06	0.43 ± 0.11	6.6E-05	0.48 ± 0.1	2.4E-06	0.35 ± 0.08	1.3E-05
WISPER	0.75 ± 0.11	2.5E-11	0.92 ± 0.06	7.3E-51	0.76 ± 0.09	8.7E-15	0.85 ± 0.08	3.8E-26	0.62 ± 0.11	1.9E-08	0.46 ± 0.11	3.5E-05	0.54 ± 0.1	1.7E-07	0.31 ± 0.08	5.5E-05
ZFAS1	0.77 ± 0.1	5.2E-13	0.84 ± 0.08	4.5E-24	0.7 ± 0.1	1.7E-11	0.78 ± 0.09	1.3E-17	0.55 ± 0.11	6.8E-07	0.36 ± 0.1	4.2E-04	0.46 ± 0.1	3.9E-06	0.3 ± 0.08	7.4E-05

Receiver operating characteristics curve was applied to differentiate between groups. A 95% confidence interval was constructed for each AUC and two-tailed Z-critical values were used to calculate p-values, . Statistical significance was considered 0.05. DR included both NPDR and PDR. DR, diabetic retinopathy; DM, diabetes mellitus; PDR, proliferative diabetic retinopathy; NPDR, nonproliferative diabetic retinopathy.

Method:

1. Sensitivity (also called True Positive Rate - TPR) was calculated for each group [C (n=11), DM (n=10), NPDR (n=26), PDR (n=23)]. Sensitivity is the probability of testing positive given that the event (disease) is positive.

2. False Positive rate (FPR) was calculated similarly. FPR is the probability of testing positive given that the event (disease) is negative.

3. Area under the curve (AUC) was approximated by summing of all the rectangles made with two sets of consecutive TPR and FPR. Formula: middle of TPR’s multiplied by change in FPR’s.

4. To calculate p-values, a confidence interval was created for each AUC. First, q0, q1, and q2 were calculated using the above formula. Then the Standard Error (SE) was calculated with n being the sample size. Alpha was considered 0.05.

5. Two-tailed Z-critical value was calculated using NORM.S.INV() function. Using the z-values and SE’s lower and upper bounds of the 95% confidence interval was calculated.

6. P-values were calculated using P = exp(−0.717×z − 0.416×z2). [https://www.bmj.com/content/343/bmj.d2304].

### Alteration of Specific lncRNAs Phenotypes May Be Predictive of DR

Given the various lncRNA expression profiles, we wanted to further determine whether combinations of specific lncRNA phenotypes existed across control and diabetic patient sub-groups. Through Z-score calculations and by comparing the mean and standard deviations for each lncRNA expression levels in diabetic patients against controls, we found that the ANRIL+/HOTAIR+/HULC+/(H19), (MIAT)/WISPER+/ZFAS1+/(H19), and ANRIL+/HOTAIR+/HULC+/WISPER+/ZFAS1+/(H19) phenotypes were prevalent in 33.9%, 35.6%, and 30.5% of all diabetic patients (n = 59 patients), respectively (**Table 6**). Interestingly, when examining both diabetic patients with either NPDR or PDR against control patients (n = 49 patients), 34.7% of these patients presented with the ANRIL+/HOTAIR+/HULC+/(H19) phenotype, 38.8% exhibited the (MIAT)/WISPER+/ZFAS1+/(H19) phenotype, and 30.6% of these patients demonstrated the ANRIL+/HOTAIR+/HULC+/WISPER+/ZFAS1+/(H19) phenotype. Specifically, in just the NPDR group (n = 26 patients), the most prevalent phenotypes included ANRIL+/HOTAIR+/HULC+/(H19) (46.2%), (MIAT)/WISPER+/ZFAS1+/(H19) (53.8%), and ANRIL+/HOTAIR+/HULC+/WISPER+/ZFAS1+/(H19) (38.5%). While the prevalent lncRNA phenotypes in the PDR group (n = 23 patients) included ANRIL+/HOTAIR+/HULC+/(H19) (21.7%), (MIAT)/WISPER+/ZFAS1+/(H19) (21.7%) and ANRIL+/HOTAIR+/HULC+/WISPER+/ZFAS1+/(H19) (21.7%). These findings suggest that unique lncRNA phenotypes could exist for specific diabetic patients, particularly those presenting at a certain stage of DR. We further applied regression model in this analysis (**Supplementary Table 1**). However, no further improvement in the diagnostic accuracy was seen.

**Table 6:** Diagnostic performance of lncRNA of various potential combinations of lncRNAs in the Control and Patient groups.

Phenotype	Control (n=11)	All patients (n=59)	DM (n=10)	NPDR (n=26)	PDR (n=23)	NPDR+PDR (n=49) (%total; %NPDR/%PDR)
Groups of 4						
ANRIL+/HOTAIR+/HULC+/H19+	0	0	0	0	0	0
ANRIL+/HOTAIR+/HULC+/(H19)	1 (9.1%)	20 (33.9%)	3 (30%)	12 (46.2%)	5 (21.7%)	17 (34.7%; 70.6/29.4)
(ANRIL)/(HOTAIR)/(HULC)/(H19)	8 (72.7%)	17 (28.8%)	3 (30%)	3 (11.5%)	11 (47.8%)	14 (28.6%; 21.4/78.6)
Groups of 4						
MIAT+/WISPER+/ZFAS1+/H19+	0	0	0	0	0	0
(MIAT)/WISPER+/ZFAS1+/(H19)	1 (9.1%)	21 (35.6%)	2 (20%)	14 (53.8%)	5 (21.7%)	19 (38.8%; 73.7/26.3)
(MIAT)/(WISPER)/(ZFAS1)/(H19)	8 (72.7%)	19 (32.2%)	5 (50%)	5 (19.2%)	9 (39.1%)	14 (28.6%; 35.7/64.3)
Groups of 6						
ANRIL+/HOTAIR+/HULC+/WISPER+/ZFAS1+/H19+	0	0	0	0	0	0
ANRIL+/HOTAIR+/HULC+/WISPER+/ZFAS1+/(H19)	1 (9.1%)	18 (30.5%)	3 (30%)	10 (38.5%)	5 (21.7%)	15 (30.6%; 66.7/33.3)
(ANRIL)/(HOTAIR)/(HULC)/(WISPER)/(ZFAS1)/(H19)	8 (72.7%)	14 (23.7%)	3 (30%)	2 (7.7%)	9 (39.1%)	11 (22.4%; 18.2/81.8)

+ means at least 1SD higher fold of lncRNA expression compared to control’s mean. lncRNA in parenthesis indicate level of lncRNA could not fall below or rise above 1SD of control’s mean. Abbreviations: DR, diabetic retinopathy; DM, diabetes mellitus; PDR, proliferative diabetic retinopathy; NPDR, nonproliferative diabetic retinopathy.

Method:

1. Mean and SD of the control (n=11) was calculated for each of the nine lncRNA.

2. A Z-score was given to each sample based on their distance from the mean and SD of the control.

3. The scores were grouped intro +1SD, +2SD +3SD or more, and -1SD.

4. Phenotypes were formed. ANRIL+ means if ANRIL is expressed at least 1SD above the control’s mean calculated in step 1. (ANRIL) means the level of ANRIL could not fall or rise above 1SD of control’s mean: -1SD < ANRIL < +1SD.

5. The date is shown in this format: #count (%percent) unless the value is 0.

6. Only column NPDR+PDR contains extra information for the percentage of each NPDR and PDR showing the phenotype separately. The first value shows % of NPDR (% of NPDR, % of PDR).

## Discussion

The emergence of diabetes as a global epidemic is a major challenge to human health in the 21^st^ century, with a global prevalence of 8.8% of the global adult population in 2017 (20). Among the diabetic population, a recent meta-analysis in 2020 estimated that the global prevalence for DR was 22.27%, 6.17% for vision-threatening DR, and 4.07% for clinically significant macular edema (21). If the current rate of DR prevalence remains constant over the next 25 years, it is estimated that the global number of adults with DR will further increase by 25.9% (129.84 million) in 2030 and by 55.6% (160.50 million) in 2045 (21). Although the likelihood of DR-induced vision impairment can be greatly reduced if DR is diagnosed in its early stages and treated accordingly, nearly one-third of patients with diabetes fail to follow vision care guidelines in the US and non-adherence has reached more than 60% in developing countries, which is largely attributable to the limited accessibility of ophthalmic services and elevated costs (22). Furthermore, the current gold standard for DR screening is based on clinical examinations (with various diagnostic tools), and DR is clinically diagnosed with the onset of ophthalmoscopic signs. However, since biochemical and functional defects in the eye often precede the development of vascular lesions and the onset of clinical signs (23), it is critical that novel, accurate, low-cost, and predictive screening tools are developed that can identify the best therapeutic window for patients who have not yet developed clinically evident retinopathy and still have their visual function intact—since this sub-population of diabetic patients comprise the majority and respond better to regimented therapies early on (23). Nevertheless, the current approaches for diagnostic evaluation can be labor-intensive, time-consuming, and susceptible to diagnostic variation due to clinical subjectivity (24) and the advent of novel diagnostic technologies, which provide effective clinical utility, is greatly required, as the prevalence of DR continues to rise exponentially.

Long non-coding RNAs (lncRNAs) represent a promising class of RNA molecules that can be targeted and exploited for therapeutic and diagnostic purposes. Given their limited protein-coding capacities, research over the last decade have demonstrated the versatile and critical roles of lncRNAs in cancer progression (25), neurodegenerative diseases (26), diabetes (27), and even diabetic complications such as DR (12, 14, 15, 28). To further demonstrate the importance of lncRNAs in the context of DR screening, the present study examines the diagnostic and prognostic potential of 9 circulating lncRNAs in diabetic patients with or without DR compared to non-diabetic controls. Serum samples of diabetic patients (with varying stages of DR) exhibited significant increases in the expressions of *ANRIL*, *H19*, *HOTAIR*, *HULC*, *MIAT*, *WISPER* and *ZFAS1*, while expression levels of *MALAT1* and *MEG3* did not demonstrate statistical significance when compared to controls. However, when further stratifying the diabetic patients into distinct subsets of DR and comparing the lncRNA expression levels against controls, significant differential expressions were observed for 8 out of the 9 lncRNAs except for *MEG3*. The differential expression profiles observed for the lncRNAs in this study are consistent with previous reports that have documented the de-regulated nature of lncRNAs in DR (12, 14, 15, 29–31), which further supports the diagnostic and prognostic potential of lncRNAs for DR screening. It is to be noted that in this exploratory study, we didn’t use a synthetic spike-in RNA as control. We used β-actin as internal control along with several positive controls (cell culture) and negative controls for our assays.

Although the mechanisms of action for lncRNAs continue to be elucidated, a large body of literature supports the notion that lncRNAs can govern gene expressions through various mechanisms: 1) guiding molecular complexes (e.g., chromatin-modifying enzymes) to target genomic regions (32), 2) functioning as a decoy for transcription factors (33), 3) serving as a scaffold to assist in the assembly of protein complexes (34), 4) directly enhancing the activation of neighboring genes (35), and 5) acting as a sponge that sequesters critical microRNAs (36). Although lncRNAs may exert more than one of the above mechanisms, the subcellular localization of lncRNAs can further provide insights into the structural features and distinct functionalities of lncRNAs, with nuclear-retained lncRNAs playing important roles in transcriptional regulation and cytoplasmic-retained lncRNAs being implicated in post-transcriptional regulation (37, 38). Furthermore, in the context of diabetes, chronic hyperglycemia can initiate various cellular events, such as angiogenesis, inflammation, oxidative damage, and fibrosis, which can further stimulate the transcription of various lncRNAs (28).

The lncRNAs used in the PCR panel are well-documented in literature. Specifically, *HOTAIR* is a trans-acting lncRNA that can directly interact with several epigenetic enzymes (e.g., polycomb repressive complex 2; PRC2) to regulate the expressions of multiple genes involved in various disease processes, including cancer progression, ischemic stroke, diabetes, and recently angiogenesis in DR (12, 29, 39, 40). As for the lncRNA *MALAT1* (also known as *NEAT2*), aberrant *MALAT1* expressions have been documented in the retinas of diabetic rodents and various retinal cells cultured in high glucose environments (31). Several studies have also demonstrated *MALAT1*’s inflammatory functionalities in various diabetic complications (14, 41, 42). Interestingly, in alignment with our findings, Shaker et al. have demonstrated that significant increases in serum *HOTAIR* and *MALAT1* levels were evident in NPDR and PDR patients compared to healthy controls and through ROC analyses, these lncRNAs could discriminate NPDR and PDR from non-DR controls, further supporting the diagnostic and prognostic utility of lncRNAs as potential non-invasive biomarkers for DR screening and early diagnosis of PDR (29).

*H19*, a conserved and maternally imprinted lncRNA, is implicated in several pathophysiological processes (43). Few studies have examined *H19* in DR, however we have previously confirmed *H19*’s role in preventing the glucose-induced phenotypic switch of endothelial cells in the diabetic retina (known as endothelial-to-mesenchymal transition; EndMT) (15). Hyperglycemia was shown to promote the upregulation of mesenchymal markers and the downregulation of both *H19* and endothelial cell markers, and subsequent overexpression of *H19* in HG-treated HRECs dramatically reversed the trends evoked by hyperglycemia, suggesting a potential protective role for *H19* in preventing EndMT in DR. Interestingly, in our current findings, *H19* expression levels were relatively increased across all diabetic patient sub-groups with or without DR compared to controls, which may be attributed to patient-specific and/or biological differences (e.g., absence of EndMT). Similar to *H19*, maternally expressed gene 3 (MEG3) is a maternally imprinted gene that exerts critical developmental properties (44). Several lines of evidence also suggest that the inactivation of this gene and the subsequent loss of the *MEG3* lncRNA (along with its diseases-suppressive properties) are frequently documented in numerous cancers and diabetic environments (19, 45–47). In parallel with our findings, reduced serum levels of *MEG3* were observed in patients with DR compared to controls, which may suggest a reduction in the protective mechanisms exerted by *MEG3* (46), however additional studies are warranted to confirm this notion.

Although the lncRNAs *WISPER* (Wisp2 super-enhancer-associated RNA), *HULC* (Highly upregulated in liver cancer), and *ZFAS1* (ZNFX Antisense RNA 1) have not been extensively examined in the context of DR, these critical lncRNAs are pathologically involved in the development of cardiac fibrosis (16), metastatic progression of hepatocellular carcinoma (48), and diabetic cardiomyopathy (17), respectively. Given the increased expressions of these lncRNAs in HG-treated HRECs and the serum and vitreous specimens of DR patients observed from our study, it may be reasonable to speculate that *WISPER*, *ZFAS1* and *HULC* may have similar pathogenetic phenotypes in patients with DR, however additional mechanistic studies are warranted to further delineate their mechanisms in DR progression.

The antisense RNA to INK4 locus (*ANRIL*; also known as CDKN2B-AS1) gene gives rise to a 3.8-kb lncRNA that is prominently deregulated in many diseases, including diabetic cardiomyopathy and nephropathy (49–51). Whether *ANRIL* exerts similar functional and mechanistic capabilities in DR remains limited. However, a recent study, demonstrated for the first time by us, alludes to the angiogenic capabilities of this lncRNA in advancing DR. As evident by the findings from both *in vitro* and *in vivo* experiments, hyperglycemia can significantly induce the upregulation of *ANRIL* in HG-treated HRECs and in the retinas of diabetic mice, and subsequent knockdown of *ANRIL* can greatly hamper glucose-induced retinal angiogenesis (13). Furthermore, in a separate study by Toraih et al., *ANRIL* and 3 additional circulating lncRNAs (*RNCR2*, *MALAT1*, and *PVT1*) did not show an association with DR progression or anti-VEGF therapy response, but the expression patterns of these lncRNAs demonstrated good diagnostic performance in differentiating DM from controls and DR (30). Another prominent lncRNA involved in the pathogenesis of DR is myocardial infarction-associated transcript (*MIAT*; also referred to as RNCR2, Gomafu, or AK028326) (11, 52). *MIAT* is significantly upregulated in the retinas of diabetic rodents, and in the fibrovascular membranes of diabetic patients compared to non-diabetic controls (11). *In vitro* experiments also indicated that expressions of *MIAT* are upregulated in several HG-treated retinal cell lines, while increased plasma levels of *MIAT* have been found to be significantly associated with the presence of DR (53). Taken together, the increased *MIAT* and *ANRIL* expressions observed in the present study confirms the pathogenetic nature of these lncRNAs documented in previous studies, and long-term clinical studies are required to further discern the diagnostic performance of these lncRNAs.

Undoubtedly, the results from the current study provide unique insights into the potential discriminative abilities of lncRNAs for identifying patients with DR from patients without DR. However, given the small sample size and cross-sectional nature of the study, additional modelling, and extrapolation of long-term outcomes between lncRNA expression levels, diabetes mellitus, and the various subsets of DR could not be determined. As well, due to the exploratory nature of this study, selection bias was inevitably introduced with respect to specimen selection. Future prospective screening studies, with larger sample sizes, should be conducted in order to increase the sensitivity of the assay and help determine whether certain sub-stages of DR can be detected, through lncRNA expression levels and/or combination of lncRNA phenotypes, before clinical diagnosis.

Nevertheless, the on-going research work on lncRNAs continues to evolve our understanding of various pathologies and their respective molecular mechanisms. Given the critical nature of lncRNAs and their involvement in disease progression, exploiting these RNA transcripts for diagnostic and/or therapeutic approaches may guide optimal treatment strategies and long-term benefits for patients. As such, the collective findings from this study highlight unique expression patterns for a subset of 9 well-studied lncRNAs (*MALAT1*, *HOTAIR*, *ANRIL*, *ZFAS1*, *HULC*, *H19*, *WISPER*, *MIAT*, and *MEG3*) for patients with and without DR, and subsequent analysis of these lncRNA signatures may provide important clinical utility for the prediction and diagnosis of DR severity. However, additional long-term and larger prospective studies are warranted in order to determine such utility of lncRNA biomarkers.

## Data Availability Statement

The original contributions presented in the study are included in the article/**Supplementary Materials**. Further inquiries can be directed to the corresponding author.

## Ethics Statement

The studies involving human participants were reviewed and approved by Western Research Ethics Board and Lawson Health Research Institute at the University of Western Ontario (London, ON, CAN). The patients/participants provided their written informed consent to participate in this study.

## Author Contributions

SC conceived, designed, and directed the study. ShC and SB performed the *in vitro* experiments. SB, AC, MG, ShC, and SC collected clinical samples and performed data analyses. SC and JG provided reagents, materials, and analysis tools. SB, AC, and SC wrote the manuscript. All authors contributed to the article and approved the submitted version.

## Funding

The Canadian Institutes of Health Research funded and supported this research.

## Conflict of Interest

The authors declare that the research was conducted in the absence of any commercial or financial relationships that could be construed as a potential conflict of interest.

## Publisher’s Note

All claims expressed in this article are solely those of the authors and do not necessarily represent those of their affiliated organizations, or those of the publisher, the editors and the reviewers. Any product that may be evaluated in this article, or claim that may be made by its manufacturer, is not guaranteed or endorsed by the publisher.

## Abbreviations

DR, diabetic retinopathy; lncRNA, long non-coding RNA; DM, diabetes mellitus; ECs, endothelial cells; NPDR, non-proliferative DR; PDR, proliferative DR; VEGF, vascular endothelial growth factor; MALAT1, metastasis associated lung adenocarcinoma transcript 1; HOTAIR, HOX transcript anti-sense RNA; H19, H19 imprinted maternally expressed transcript; WISPER, Wisp2 super-enhancer-associated RNA; ZFAS1, ZNFX1 antisense RNA 1; MIAT, myocardial infarction-associated transcript; HULC, highly up-regulated in liver cancer; ANRIL, antisense non-coding RNA in the INK4 locus; MEG3, maternally expressed gene 3; NG, normoglycemia; HG, hyperglycemia or high glucose; HRECs, human retinal microvascular endothelial cells.
