# Supplementary material for: Expressions of Serum lncRNAs in Diabetic Retinopathy – A Potential Diagnostic Tool
Source: Front Endocrinol (Lausanne). 2022 Apr 7;13:851967. doi: 10.3389/fendo.2022.851967 (PMC9022211; doi:10.3389/fendo.2022.851967)
Supplement: Supplementary file 1 [file Presentation_1.pptx]

## Slide 1
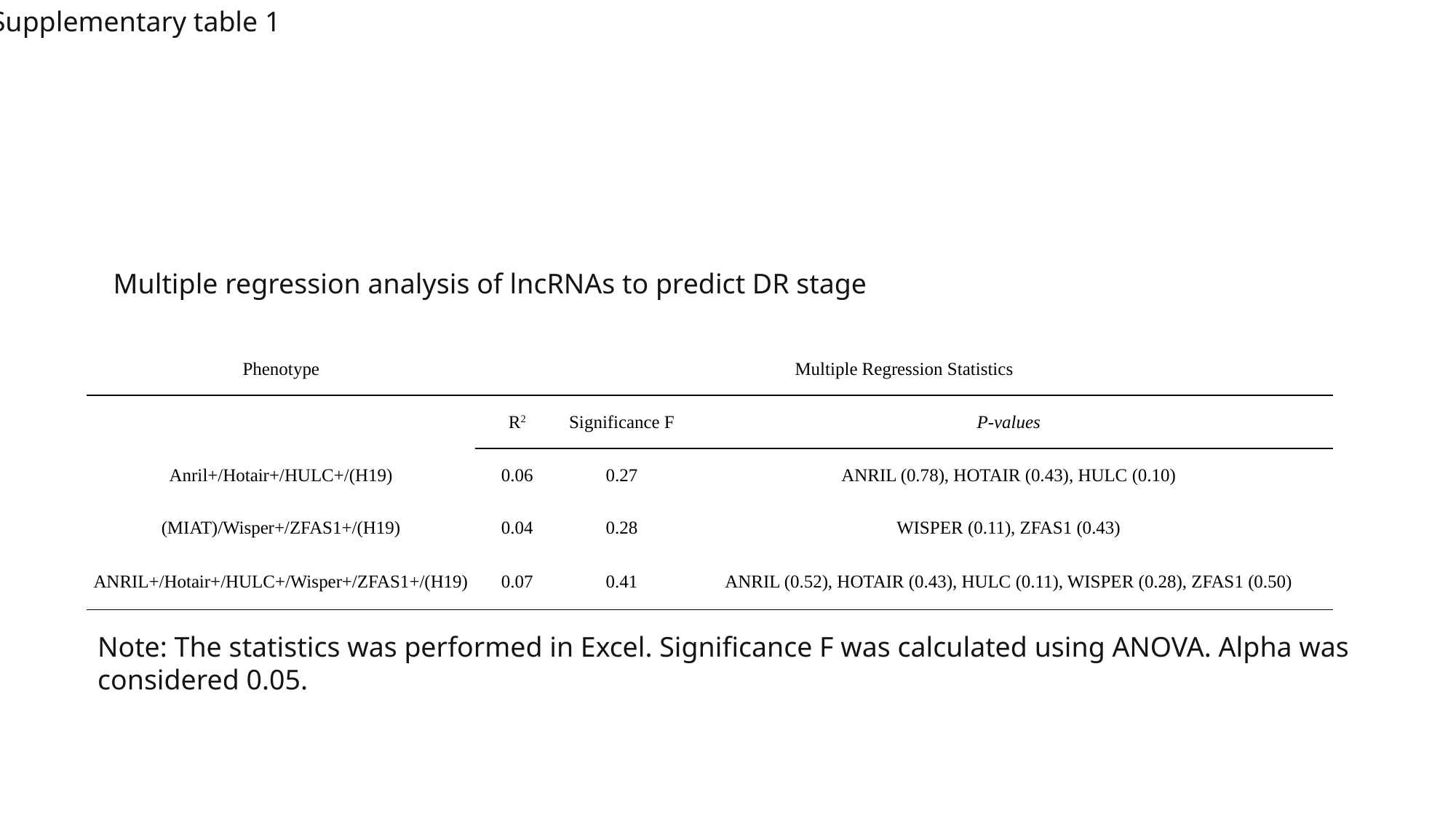

Supplementary table 1
Multiple regression analysis of lncRNAs to predict DR stage
| Phenotype | Multiple Regression Statistics | | |
| --- | --- | --- | --- |
| | R2 | Significance F | P-values |
| Anril+/Hotair+/HULC+/(H19) | 0.06 | 0.27 | ANRIL (0.78), HOTAIR (0.43), HULC (0.10) |
| (MIAT)/Wisper+/ZFAS1+/(H19) | 0.04 | 0.28 | WISPER (0.11), ZFAS1 (0.43) |
| ANRIL+/Hotair+/HULC+/Wisper+/ZFAS1+/(H19) | 0.07 | 0.41 | ANRIL (0.52), HOTAIR (0.43), HULC (0.11), WISPER (0.28), ZFAS1 (0.50) |
Note: The statistics was performed in Excel. Significance F was calculated using ANOVA. Alpha was considered 0.05.
